# *Inside CKD*: a microsimulation modelling study projects the clinical and economic burden of chronic kidney disease in Hungary

**DOI:** 10.3389/fneph.2024.1458607

**Published:** 2024-10-18

**Authors:** Lilla Szabó, Luca Adél Halmai, Erzsébet Ladányi, Juan Jose Garcia Sanchez, Salvatore Barone, Claudia Cabrera, Lise Retat, Laura Webber, István Wittmann, Boglárka Laczy

**Affiliations:** ^1^ Medical and Market Access, AstraZeneca Ltd., Budapest, Hungary; ^2^ Tritonlife Nephrology Center, Miskolc, Hungary; ^3^ Global Health Economics, BioPharmaceuticals, AstraZeneca, Cambridge, United Kingdom; ^4^ Global Medical Affairs, BioPharmaceuticals AstraZeneca, Gaithersburg, MD, United States; ^5^ Real World Science and Analytics, BioPharmaceuticals Medical, AstraZeneca, Gothenburg, Sweden; ^6^ HealthLumen Ltd, London, United Kingdom; ^7^ Second Department of Medicine and Nephrology-Diabetes Center, University of Pécs Medical School, Pécs, Hungary

**Keywords:** chronic kidney disease, disease burden, economic burden, Hungary, *Inside CKD*

## Abstract

**Objectives:**

The *Inside CKD* programme implemented a microsimulation modelling approach to project the clinical and economic burden of chronic kidney disease (CKD) between 2024 and 2027 in Hungary.

**Methods:**

Using the peer-reviewed *Inside CKD* microsimulation, a virtual Hungarian population was generated that was derived from national records, local demographic data and published epidemiological data. These inputs defined the likelihood of a change in health state for each individual as they progressed through the model in annual increments. Individual CKD status, including disease progression, cardiorenal complications and associated costs, was tracked annually to generate the population-level projections of the clinical and economic burden of CKD.

**Results:**

By 2027, people with CKD were projected to constitute 13.3% of the Hungarian national population. The prevalence of heart failure, myocardial infarction and stroke in people with CKD were projected to remain consistently high, reaching 323 447, 69 188 and 120 118 by 2027, respectively. Kidney replacement therapy cases were predicted to remain high at 20 515 in 2024 and 22 325 in 2027, with associated costs increasing from 71.4 billion HUF in 2024 to 79.6 billion HUF in 2027. Total annual healthcare costs associated with treating CKD were projected to constitute 5.4% of the overall national healthcare budget in 2027.

**Conclusions:**

*Inside CKD* demonstrates that the future burden of CKD in Hungary will be substantial unless current management strategies change. The high prevalence of undiagnosed CKD and associated cardiorenal complications highlight the urgent need for policy interventions focused on early diagnosis and timely intervention to mitigate the future burden of CKD.

## Introduction

Chronic kidney disease (CKD) is a serious, progressive condition identified through abnormalities of kidney structure and/or function that persist for over 3 months. Two tests are used to diagnose CKD; a reduced estimated glomerular filtration rate (eGFR) of < 60 mL/min/1·73 m^2^ indicates impaired kidney function, and an elevated albumin excretion rate (AER) of > 30 mg/g creatinine indicates kidney damage (1–3). CKD is classified by the level of albuminuria (stages A1–3), and the reduction of eGFR (stages G1–5). These factors are critical for charting the course of the disease and, in recognition of this, the Kidney Disease: Improving Global Outcomes (KDIGO) Expert Working Group established a CKD assessment system to diagnose and classify patients by disease stages (2). This approach uses a composite of eGFR and AER values, in which stages increase in severity from 1 to 5 (1–4); in the early stages, patients gradually lose normal kidney function but often remain asymptomatic, so may be initially unaware of their illness (1). Patients in the latter stages are more likely to experience kidney failure and to require kidney replacement therapy (KRT) (3–6).

The different stages of CKD present different challenges for policymakers and clinicians: patients in the milder stages of the disease may have their disease undiagnosed, and so they do not receive any medication or care. By stages 4 and 5, treatment is intensive and invasive, and healthcare resources must be allocated efficiently for effective disease management. These considerations are important given the high prevalence of CKD: current estimates suggest that it affects 9.1–13.4% of the population worldwide and broadly correlates with age, with approximately one-third of patients over 70 years old (1, 3, 7). Prevalence is expected to increase in the future as a result of increased life expectancy (5), but age is not the only driver of CKD, and several common conditions also act as risk factors, such as diabetes and hypertension (3, 4). CKD is also considered a risk factor for cardiovascular complications such as heart failure (HF), myocardial infarction (MI) and stroke, as well as increasing the overall risk of mortality. As a result of these associations, patients may have multiple chronic diseases that exacerbate the clinical impact of CKD and complicate healthcare strategies, especially in settings where resources may be scarce (3).

These trends are broadly consistent for Hungary, where a prevalence study based on medical records from 2011 to 2019 provided insight into the national picture. These data were used to estimate that 14.0% of the population had CKD, a relatively high frequency compared with estimates for the global population (1, 3, 8). The study also found relatively substantial rates of comorbid conditions and complications in the diagnosed CKD population, namely, diabetes (41.5%); hypertension (70.2%); HF (20.5%); stroke (10.5%); and MI (9.4%) (8). When considering prevalence data for Hungary, it is notable that the country has sustained a persistent decline in its population for four decades; census data suggest a decrease of over 1 million people has occurred in this timeframe, with the United Nations estimating a population decrease of as much as one-third by 2070 (9). This means that a persistently high proportion of the population of Hungary may have CKD, even if, in numerical terms, the number of cases falls as the overall population declines.

Although these findings usefully illustrate the scale of the issue, they can only reflect the situation during this particular time period in Hungary; optimising policy requires anticipation of future epidemiological trends. Predictions can be enhanced by complementing current epidemiological studies with simulations in which models project the long-term scope of a given disease at both the population level and the individual level. In some cases, these simulations can be further used to predict the outcomes associated with different policy interventions (10). The use of microsimulations in particular can provide valuable insights for evaluating the impact of interventions in a real-world setting, because they can take account of the risk factors and predict how these may change over time.

*Inside CKD* uses a microsimulation model of a virtual population based on known demographic, epidemiological and economic data sources to analyse the projected prevalence and burden of CKD. It can also account for the impact of national-level interventions, such as screening programmes, to raise disease awareness and assess potential strategies (10). The objective of this study was to adapt the microsimulation modelling approach of *Inside CKD* to a Hungarian setting to project the national clinical and economic burden of CKD between 2024 and 2027.

## Methods

The *Inside CKD* research programme developed a microsimulation model to quantify the future clinical and economic burden of CKD, and to assess the potential long-term impact of different interventions.

A detailed description of the model that was initially tested using a UK case study has already been published (10). In brief, the *Inside CKD* microsimulation generated a hypothetical cohort of 20 million individuals aged between 0 and 110 years. This virtual population was based on predefined demographic, epidemiological and economic data sources specific to Hungary. Each individual in the modelled population was assigned demographic characteristics (age and sex), and baseline eGFR and AER values; these criteria allowed the model to classify individuals with a CKD status (no CKD or CKD stages 1–5). CKD stages were based on the severity of the disease and comprised a composite of AER and eGFR, as described in the KDIGO guidelines (**Supplementary Table 1**) (2). For the population with CKD, current diagnosis rates were used to estimate the proportion who were undiagnosed. The likelihood of developing CKD (in the healthy population), the worsening of eGFR and AER values (signifying disease progression in the CKD population), and other clinical and economic parameters were modelled annually over the 4-year simulation (**Supplementary Figure 1**). Individual clinical characteristics and regression analyses were based on the DISCOVER CKD database and used to generate estimates of disease progression based on eGFR and AER values (11).

To evaluate the robustness of the model, sensitivity analyses of the key parameters (e.g. the impact of changes in assumptions, discount rates and the use of proxy data on outcomes) were performed on the UK case study (10). Parametric uncertainty was analysed for the UK case study based on eGFR slopes, relative risks, KRT and diagnosis rates. The following validity types were assessed: face validity, internal validity, external validity, predictive validity and cross-validity; these have been recently published (10, 12, 13). For Hungary, the sensitivity analysis examined the impact of a 10% increase and a 10% decrease in diagnosis rates on CKD prevalence (**Supplementary Figure 2**).

In this study, Hungary-specific data were used to generate local inputs: population, disease burden, CKD status, eGFR slopes, KRT and costs (**Supplementary Table 2**). For the clinical and economic burden of CKD, a time frame from 2024 to 2027 was used and the analysis was carried out from the national healthcare perspective of Hungary.

### Input variables

General population data, categorised by sex and age, were derived using entries for Hungary from a United Nations database (9), which also provided estimates for fertility rates and live births (9). Mortality was based on data from the Human Mortality Database for Hungary (14). Clinical input variables were derived from the real-world financing database of the Hungarian National Health Insurance Fund (NHIF) and were based on a 5.5-year study period (2016–2021). The NHIF database covers almost all of the Hungarian population; in the analysis, the majority of the Hungarian population was assumed to have access to healthcare (94%) and/or to be registered with a primary care physician (99%) (15, 16). The database allowed retrospective analysis of longitudinal data, which were available as aggregated outputs. The prevalence of comorbid conditions and complications (namely, diabetes, hypertension, HF, stroke and MI) were also determined from this database. The frequency, cost and modality of KRT were sourced from the Hungarian Society of Nephrology (MANET) database (2016–2019) (17), the NHIF database, and a comprehensive publication by O’Callaghan and colleagues (18). The MANET database included patients treated in one of the dialysis centres in Hungary, and only patients with chronic disease were included (**Supplementary Table 2**).

Costs were taken from NHIF turnover data. Outpatient costs were characterised using International Statistical Classification of Diseases (that included reimbursement costs) and Related Health Problems codes, and inpatient care costs were based on diagnosis-related groups. All costs were presented in Hungarian forint (HUF). All financial estimates were modelled in accordance with the Hungarian national healthcare system before validation by local experts. These financial estimates have been previously reported in the published *Inside CKD* cost library (19) and additional detail is provided in **Supplementary Table 2**.

Hungary-specific data were not always available for a specific input variable; in these cases, proxy data from other countries with similar demographic or healthcare profiles were used as follows: eGFR and albuminuria rates, data from Poland; KRT initiation thresholds and treatment probability, data from the UK; prevalence of transplantation, type 2 diabetes and hypertension, data from Romania; and diagnosis rates, data from the UK and USA. Some of the Hungarian cardiovascular complication data were also supplemented with US findings. Proxies were chosen based on an algorithm that assessed comparability of epidemiology between countries with available data and Hungary, and were further refined by a scientific steering committee that oversaw clinical and protocol decisions for the *Inside CKD* project (10).

### Output variables

For the clinical burden, we estimated the projected prevalence of CKD by stage and for diagnosed versus undiagnosed individuals, comorbidity status, the presence of cardiovascular complications, KRT frequency and modality, and all-cause mortality. Direct costs for patients with diagnosed CKD were estimated using local literature and databases, and were product agnostic. Indirect costs, hospitalisation costs and medication were not included, but treatment costs of KRT (haemodialysis, peritoneal dialysis, and transplantation surgery and maintenance) and costs associated with HF, MI, and stroke were included.

## Results

### Disease burden of CKD in Hungary

**Figure 1:**
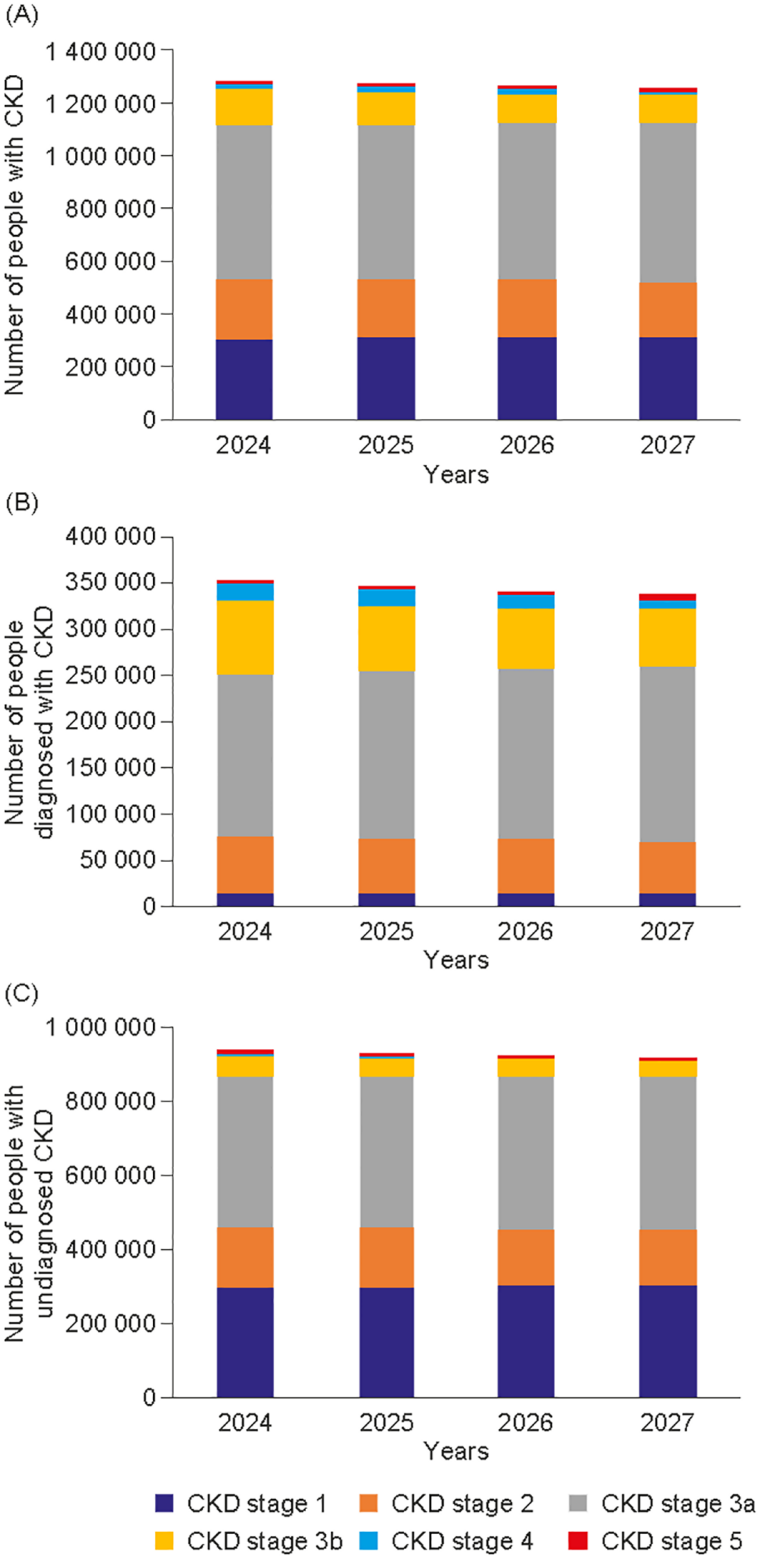
Projected prevalence, total cases **(A)**, undiagnosed cases **(B)** and diagnosed cases **(C)** by chronic kidney disease stage in Hungary from 2024 to 2027. CKD stage 5 includes patients undergoing kidney replacement therapy. Diagnosed CKD represents people with a medical record of the condition. CKD, chronic kidney disease.

**Figure 2:**
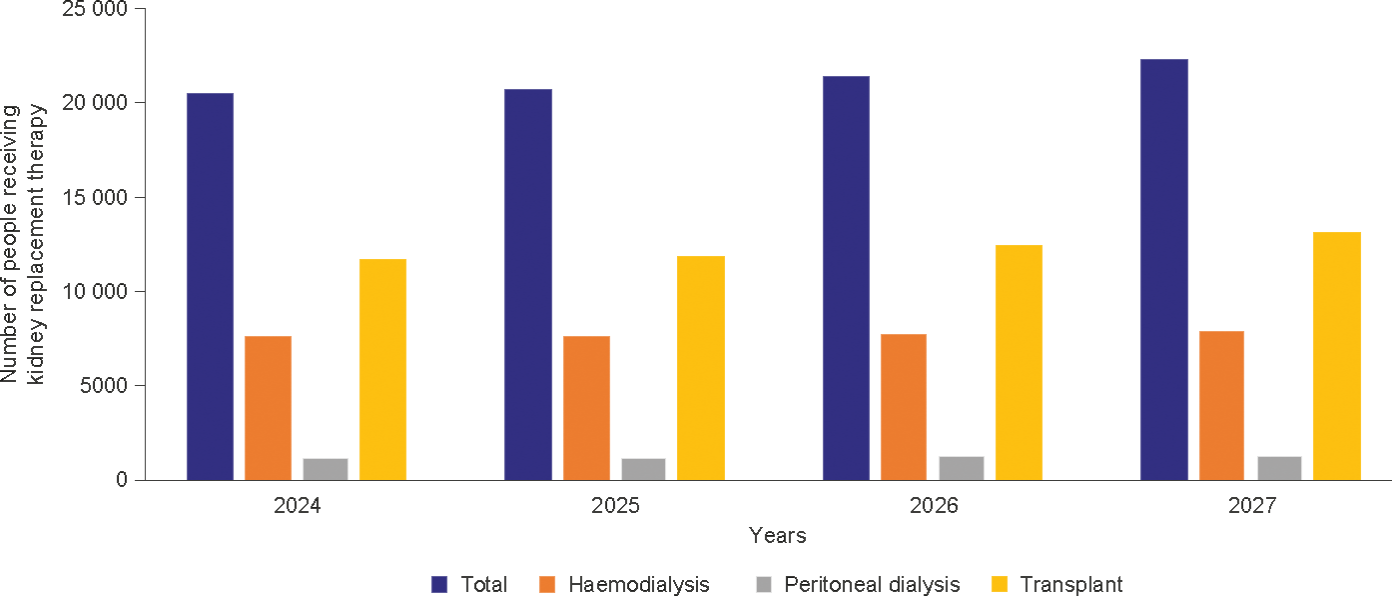
Projected use of kidney replacement treatment by modality. These data represent the diagnosed population of people with CKD. CKD, chronic kidney disease.

In our simulation, the number of people with CKD was projected to decrease from 1.28 million individuals in 2024 to 1.25 million in 2027, but the relative proportion of the overall population was projected to remain stable and high (13.4% in 2024, 13.3% in 2027). When younger patients were excluded, adult patients with CKD (≥ 18 years old) were projected to constitute 15.8% of the overall population by 2027. Of the people predicted to have CKD in 2027, the model estimated that only 27.0% (0.3 million) would have a formal diagnosis. The distribution of CKD stages varied, with diagnoses proportionately higher in later stages of CKD (**Figure 1**). The use of KRT was projected to increase from 20 515 in 2024 to 22 325 by 2027. In terms of modality, by 2027, 7911 (35.4%) people were projected to require haemodialysis, 1250 (5.6%) to need peritoneal dialysis, and 13 164 (59.0%) to require a kidney transplant (**Figure 2**).

**Figure 3:**
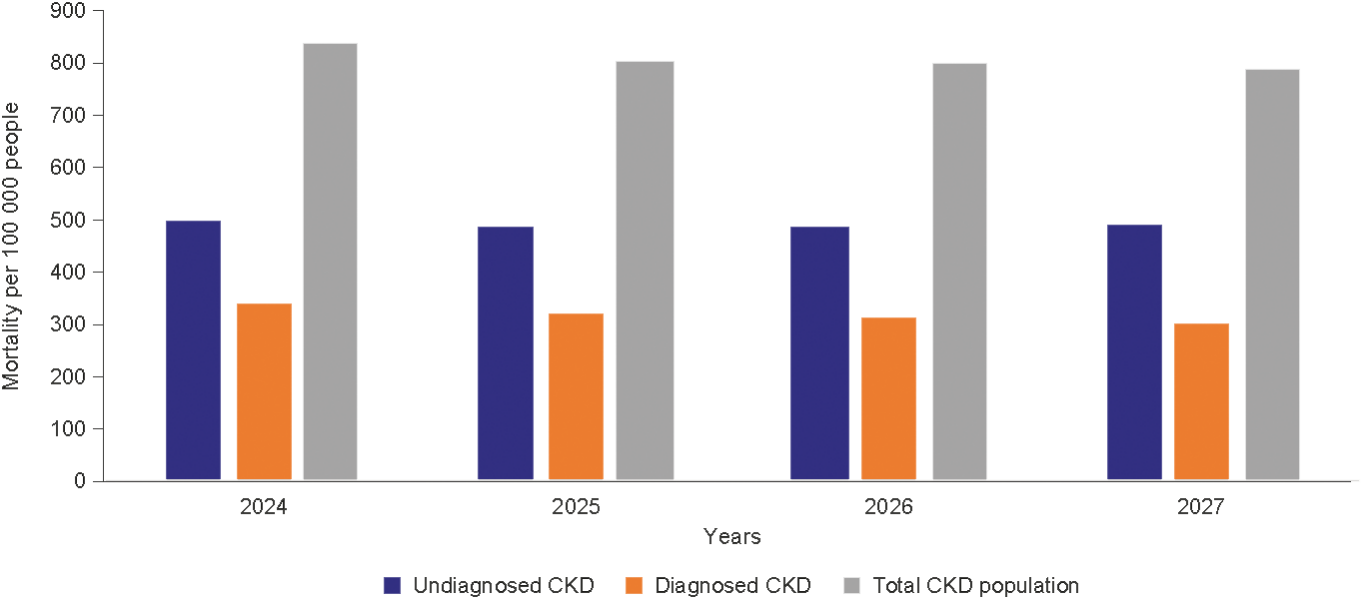
Projected mortality by chronic kidney disease diagnosis status. Diagnosed CKD represents people with a medical record of the condition. CKD, chronic kidney disease.

In the microsimulation timeframe (2024–2027), cardiovascular complications for HF, MI, and stroke were projected to remain high and stable across all CKD stages, reaching an overall prevalence of 323 447, 69 188 and 120 118 people, respectively, by 2027 (**Table 1**). Most of these cases of HF, MI and stroke were estimated to occur in patients with CKD stage 3 (n = 239 657, 51 944 and 86 642, respectively), reflecting the size of this population relative to the populations of other stages. Comorbidities commonly associated with CKD – namely, hypertension and type 2 diabetes – were projected at consistently high rates, which were maintained across the simulation time period (**Table 2**). By 2027, within the CKD population 657 007 people were projected to also have hypertension, 92 824 to have type 2 diabetes and 250 258 to have both conditions. Annual mortality was highest in the population of people with undiagnosed CKD (n = 913 707 versus 338 710 in the diagnosed population in 2027). The risk of death was predicted to be highest in the later stages of CKD, although in terms of absolute numbers, the burden was predicted to be highest in CKD stage 3a and stage 3b relative to other stages, again owing to a larger overall number of patients in this category (**Figure 3**).

**Table 1:** Projected prevalence of cardiovascular complications by chronic kidney disease stage.

		Heart failure		Myocardial infarction		Stroke	
		2024	2027	2024	2027	2024	2027
Prevalence per 100 000	Stage 1	1685	2036	327	399	579	712
	Stage 2	4250	4225	906	887	1779	1777
	Stage 3a	14 684	15 453	3177	3336	5290	5531
	Stage 3b	4705	3682	1035	812	1782	1387
	Stage 4	560	276	117	64	229	125
	Stage 5	75	154	16	27	33	59

Population given as a proportion of people with diagnosed chronic kidney disease.

**Table 2:** Projected prevalence of comorbidities in people with chronic kidney disease.

		Hypertension only		Type 2 diabetes only		Type 2 diabetes and hypertension	
		2024	2027	2024	2027	2024	2027
Prevalence per 100 000	Stage 1	52 029	51 928	7520	7528	24 338	24 420
	Stage 2	51 874	51 594	7540	7644	24 647	24 850
	Stage 3a	52 316	52 530	7548	7519	17 496	17 096
	Stage 3b	52 833	55 145	6734	6134	15 732	13 949
	Stage 4	53 265	56 441	6649	5442	15 956	14 313
	Stage 5	50 766	47 663	7311	9063	16 975	20 948

Population given as a proportion of people with diagnosed CKD; people with CKD may also have had other comorbidities that were not factored into the virtual scenario. CKD, chronic kidney disease.

### Economic burden of CKD in Hungary

The total annual healthcare costs associated with diagnosed CKD and excluding KRT were likely to remain high, at 54.0 billion HUF in 2024 and 51.4 billion HUF in 2027. However, the cost of KRT was projected to be 71.4 billion HUF in 2024, increasing to 79.6 billion HUF in 2027, illustrating the impact of disease stage and kidney failure on costs. The proportion of total annual healthcare expenditure in Hungary spent on diagnosed CKD was projected to reach 5.4% by 2027, most of which comprised KRT costs (1.7% for a kidney transplant, 1.5% for haemodialysis and 0.2% for peritoneal dialysis). Complications arising from CKD were estimated at 2.7 billion HUF for HF, 8.4 billion HUF for MI and 20.1 billion HUF for stroke in 2024, with the equivalent values for 2027 at 2.5 billion HUF for HF, 7.8 billion HUF for MI and 18.5 billion HUF for stroke.

### Sensitivity analysis

As diagnosis rates were not available for Hungary, a US proxy was used in the analyses. Therefore, the effect of the proxy on the results was assessed in a sensitivity analysis in which CKD prevalence was projected using a 10% increase and decrease in diagnosis rates. When the analysis was run, diagnosed cases were projected to reach between 0.23 million and 0.45 million cases by 2027.

## Discussion

Our analysis projects a substantial burden of CKD in Hungary, underscoring the need to identify solutions to mitigate the clinical and economic effects of the disease. Although the *Inside CKD* programme takes a global perspective, the importance of considering projecting data at the national level is a critical aspect of raising awareness and stimulating political debate on the value of healthcare strategies.

The population of Hungary has been in decline since 1981, when the population size was 10 711 848; current estimates for 2023 suggest a figure of 9 599 744 (20). This general population trend explains the decrease from 1.28 million cases of CKD to 1.25 million cases of CKD. However, the overall proportion of people with the condition will remain high at approximately 13%, suggesting that the disease will continue to pose a substantial challenge and, in relative terms, that prevalence remains stable. When only the adult population (≥ 18 years old) is considered, patients with CKD are projected to constitute 15.8% of the population by 2027, notably higher than the prevalence observed for other *Inside CKD* regions (12). This observation may partially be explained by the prevalence of underlying risk factors that predispose individuals to CKD: Hungary has comparatively high rates of hypertension (21), coronary heart disease (22), stroke (22), smoking (23) and obesity (24). Additionally, the proportion of the population aged 65 years and over is relatively high for Hungary compared with other *Inside CKD* regions (25).

The projected CKD population consisted of diagnosed and undiagnosed patients, with the latter category comprising the majority of this population: nearly two-thirds of people with CKD in 2027 are projected not to have received a diagnosis. Corresponding global data show a similar trend of under-recognition and undertreatment (1), and even those with CKD stage 3, when symptoms may become apparent, remain undiagnosed in 61.6–95.5% of cases (5). Early detection of CKD is an obvious prerequisite of treatment that can, if given appropriately, delay disease progression. Timely therapeutic intervention may mitigate long-term morbidity and mortality and may delay the development of kidney failure (3). This may be particularly relevant to the Hungarian healthcare context given the disproportionate impact of late-stage CKD on resources. In our Hungarian data set, patients receiving KRT were projected to constitute a relatively small population group compared with those of earlier stages of CKD, yet they had the highest relative financial burden. Given that KRT cases were projected to increase despite general population trends, policymakers may assume that budget allocation for this demographic will remain high. Disease progression is also associated with an increased risk of cardiorenal symptoms, which affect quality of life and increase the risk of mortality. The latter in particular, is concerning, given that the Global Burden of Disease study found that the all-age mortality due to CKD increased by 41.5% during the analysed period (3) and our findings broadly corroborate these results, although it should be noted that deaths cannot be attributed solely to CKD.

Although the microsimulation provided projections up to 2027, we can hypothesise that this population of patients with moderate disease may progress to the more severe category if effective treatments are not used, driving costs up further. If no changes are made to the current management of CKD, the clinical and economic burden will be substantial. Early treatment – when appropriate – may have a crucial role in protracting the progression of CKD and delaying, or even preventing, the need for invasive KRT strategies. Such interventions are dependent on diagnosis, which is unacceptably low in the current paradigm. The benefits of an early diagnosis include the opportunity for treatment, the monitoring of other, associated health complications and potential comorbidities, and the empowerment of the patient to make lifestyle and therapeutic decisions.

As with any simulation, the data generated were heavily dependent on the input variables and assumptions used to construct the model. In terms of costs, hospitalisation costs were excluded and costs associated with CKD stages 1 and 2 were assumed to be zero; in both cases, this was because of potential confounding with other conditions that might lead to an overestimation. Costs were calculated for patients with diagnosed CKD only, because undiagnosed patients are likely to be untreated, and indirect costs could not be accounted for owing to a lack of suitable economic data for modelling. Combining these assumptions, it is likely that our financial costs are conservative; however, the costs for complications were potentially accounted for in the costs for CKD, so they may have been slightly overestimated. In terms of clinical limitations, the use of proxy data in the absence of published or registry data for Hungary may have introduced uncertainty into the simulation; however, given the flexible nature of the microsimulation, it can be re-run to include more accurate data as new epidemiological research emerges. Notably, the use of registry data for Hungary to capture more specific input data may improve accuracy. Regarding model uncertainty, a comprehensive, stochastic, parametric uncertainty analysis is unsuitable for large-scale non-linear models, such as the *Inside CKD* microsimulation. Therefore, a global sensitivity analysis was carried out on diagnosis rates (**Supplementary Figure 2**), which is a validated approach for assessing the parametric uncertainty for individual parameters (26). Finally, the simulations did not account for the impact of the COVID-19 pandemic and sociopolitical events that may have indirectly affected healthcare was not accounted for. Again, future simulations may enable modelling of these events and other significant variables.

The projected clinical and economic burden demonstrates the necessity of policy interventions. Targeted policies could be orientated towards risk factors for CKD, addressing the underlying factors that may be driving prevalence. For example, food reformulation programmes have been successfully used to reduce salt in processed foods, a known risk factor for hypertension (27, 28). More direct interventions could include screening at-risk patients, such as those with type 2 diabetes or hypertension (29), which would facilitate the diagnosis of patients with early-stage CKD and enable the earlier use of medications. An effective programme would rely on cooperation between primary and secondary care and would support communication and cooperation between primary care physicians, cardiologists, diabetologists and hypertension experts (1).

Our study corroborates existing concerns regarding the scale of CKD and, in particular, its impact on healthcare systems. By exploring the data for a single country (Hungary), we were able to consider these data in a national context, quantifying and projecting key epidemiological data into the near future. We demonstrated a stable, persistent and high prevalence of CKD in the general population, associated with a high prevalence of cardiovascular complications and mortality, as well as extensive treatment costs. If no remedial action is taken proactively to manage the drivers and progression of CKD, the future disease burden will be considerable. Clinical inertia and stagnation need to be challenged with active measures to identify, diagnose and treat patients with CKD.

## Data Availability

The original contributions presented in the study are included in the article/**Supplementary Material**. Further inquiries can be directed to the corresponding author/s.

## References

[1] Erzsébet L. Krónikus veseelégtelenség korai diagnosztikájának jelentősége. Hypertonia és Nephrol. (2023) 27:23–8. doi: 10.33668/hn.27.005

[2] Kidney Disease: Improving Global Outcomes C. K. D. Work Group. KDIGO 2024 clinical practice guideline for the evaluation and management of chronic kidney disease. Kidney Int. (2024) 105:S117–314. doi: 10.1016/j.kint.2023.10.018 38490803

[3] Global Burden of Disease Chronic Kidney Disease Collaboration. Global, regional, and national burden of chronic kidney disease, 1990-2017: a systematic analysis for the Global Burden of Disease Study 2017. Lancet. (2020) 395:709–33. doi: 10.1016/S0140-6736(20)30045-3 PMC7049905 32061315

[4] Levey AS Coresh J. Chronic kidney disease. Lancet. (2012) 379:165–80. doi: 10.1016/S0140-6736(11)60178-5 21840587

[5] Tangri N Moriyama T Schneider MP Virgitti JB De Nicola L Arnold M. Prevalence of undiagnosed stage 3 chronic kidney disease in France, Germany, Italy, Japan and the USA: results from the multinational observational REVEAL-CKD study. BMJ Open. (2023) 13:e067386. doi: 10.1136/bmjopen-2022-067386 PMC10230905 37217263

[6] Levey AS Eckardt KU Dorman NM Christiansen SL Cheung M Jadoul M. Nomenclature for kidney function and disease: executive summary and glossary from a Kidney Disease: Improving Global Outcomes (KDIGO) Consensus Conference. Transpl Int. (2020) 33:999–1009. doi: 10.1111/tri.13627 32337774

[7] Hill NR Fatoba ST Oke JL Hirst JA O’Callaghan CA Lasserson DS. Global prevalence of chronic kidney disease - A systematic review and meta-analysis. PloS One. (2016) 11:e0158765. doi: 10.1371/journal.pone.0158765 27383068 PMC4934905

[8] Zemplenyi A Saghy E Konyi A Szabo L Wittmann I Laczy B. Prevalence, cardiometabolic comorbidities and reporting of chronic kidney disease; A Hungarian cohort analysis. Int J Public Health. (2023) 68:1605635. doi: 10.3389/ijph.2023.1605635 37065645 PMC10101229

[9] United Nations Department of Economic and Social Affairs, Population Division. World Population Prospects 2022 (2022). Available online at: https://population.un.org/wpp/Download/Standard/Population/ (Accessed 1 July, 2022).

[10] Tangri N Chadban S Cabrera C Retat L Garcia Sanchez JJ. Projecting the epidemiological and economic impact of chronic kidney disease using patient-level microsimulation modelling: rationale and methods of inside CKD. Adv Ther. (2023) 40:265–81. doi: 10.1007/s12325-022-02353-5 PMC9616410 36307575

[11] Pecoits-Filho R James G Carrero JJ Wittbrodt E Fishbane S Sultan AA. Methods and rationale of the DISCOVER CKD global observational study. Clin Kidney J. (2021) 14:1570–8. doi: 10.1093/ckj/sfab046 PMC8264307 34249352

[12] Chadban S Arıcı M Power A Wu M-S Mennini FS Alvarez Arango JJ. Projecting the economic burden of chronic kidney disease at the patient level (Inside CKD): a microsimulation modelling study. eClinicalMedicine. (2024) 72:102615. doi: 10.1016/j.eclinm.2024.102615 39010976 PMC11247148

[13] Chertow GM Correa-Rotter R Eckardt KU Kanda E Karasik A Li G. Projecting the clinical burden of chronic kidney disease at the patient level (Inside CKD): a microsimulation modelling study. eClinicalMedicine. (2024) 72:102614. doi: 10.1016/j.eclinm.2024.102614 39010981 PMC11247147

[14] Human Mortality Database (HMD). (2021). HMD: Hungary Max Planck Institute for Demographic Research (Germany), University of California, Berkeley (USA), and French Institute for Demographic Studies (France). Available online at: https://www.mortality.org/Country/Country?cntr=HUN (Accessed August, 2021).

[15] Hivatal Központi Statisztikai. Egészségügyi beavatkozások és szolgáltatások (2004–2018) (2020). Available online at: https://www.ksh.hu/thm/2/indi2_8_3.html (Accessed July, 2021).

[16] Hivatal Központi Statisztikai. Háziorvosok és házi gyermekorvosok (2023). Available online at: https://www.ksh.hu/docs/hun/xstadat/xstadat_eves/i_fea002b.html (Accessed August, 2021).

[17] Magyar Nephrologiai Társaság. MANET: Hungarian Society of. Magyar Nephrologiai Társaság (2016–2019) (2020). Available online at: http://www.nephrologia.hu/info.aspx?sp=9 (Accessed August, 2021).

[18] O’Callaghan CA Shine B Lasserson DS. Chronic kidney disease: a large-scale population-based study of the effects of introducing the CKD-EPI formula for eGFR reporting. BMJ Open. (2011) 1:e000308. doi: 10.1136/bmjopen-2011-000308 PMC3244664 22184586

[19] Jha V Al-Ghamdi SMG Li G Wu MS Stafylas P Retat L. Global economic burden associated with chronic kidney disease: A pragmatic review of medical costs for the inside CKD research programme. Adv Ther. (2023) 40:4405–20. doi: 10.1007/s12325-023-02608-9 PMC10499937 37493856

[20] Hivatal Központi Statisztikai. 22.1.1.1. Main indicators of population and vital events (2023). Available online at: https://www.ksh.hu/stadat_files/nep/en/nep0001.html (Accessed November, 2023).

[21] World Health Organization Global Health Observatory. Global Health Observatory data repository: raised blood pressure (SBP>=140 OR DBP>=90) (age-standardized estimate) (2017). Available online at: https://www.who.int/data/gho/data/indicators/indicator-details/GHO/raised-blood-pressure-(sbp-=140-or-dbp-=90)-(age-standardized-estimate) (Accessed August, 2021).

[22] Global Burden of Disease. Global Burden of Disease Study 2019 (GBD 2019) Data Resources: ischemic heart disease, stroke (2019). Available online at: http://ghdx.healthdata.org/gbd-2019 (Accessed August, 2021).

[23] The World Bank. World Health Organization, Global Health Observatory Data Repository: prevalence of current tobacco use (% of adults) (2021). Available online at: https://data.worldbank.org/indicator/SH.PRV.SMOK (Accessed August, 2021).

[24] World Health Organization Global Health Observatory. Global Health Observatory data repository: prevalence of obesity among adults, BMI ≥ 30, age-standardized, estimates by country (2017). Available online at: https://apps.who.int/gho/data/view.main.CTRY2450A (Accessed August, 2021).

[25] World Health Organization Global Health Observatory. United Nations Population Division. World Population Prospects: population ages 65 and above (% of total population) (2021). Available online at: https://data.worldbank.org/indicator/SP.POP.65UP.TO.ZS (Accessed August, 2021).

[26] Jaccard A Retat L Brown M Webber L Chalabi Z. Global sensitivity analysis of a model simulating an individual’s health state through their lifetime. Microsimulation. (2018) 11:100–21. doi: 10.34196/ijm.00190

[27] He FJ Brinsden HC MacGregor GA. Salt reduction in the United Kingdom: a successful experiment in public health. J Hum Hypertens. (2014) 28:345–52. doi: 10.1038/jhh.2013.105 24172290

[28] He FJ Li J MacGregor GA. Effect of longer term modest salt reduction on blood pressure: Cochrane systematic review and meta-analysis of randomised trials. BMJ. (2013) 346:f1325. doi: 10.1136/bmj.f1325 23558162

[29] Shlipak MG Tummalapalli SL Boulware LE Grams ME Ix JH Jha V. The case for early identification and intervention of chronic kidney disease: conclusions from a Kidney Disease: Improving Global Outcomes (KDIGO) Controversies Conference. Kidney Int. (2021) 99:34–47. doi: 10.1016/j.kint.2020.10.012 33127436

