# Supplementary material for: Inside CKD: a microsimulation modelling study projects the clinical and economic burden of chronic kidney disease in Hungary
Source: Front Nephrol. 2024 Oct 18;4:1458607. doi: 10.3389/fneph.2024.1458607 (PMC11527778; doi:10.3389/fneph.2024.1458607)
Supplement: Supplementary file 1 [file DataSheet1.pdf]

Supplementary Material

Contents

1 Supplementary Figures and Tables....2
1.1 Supplementary Figures....2
1.2 Supplementary Tables....4
2 Supplementary references....8

1 Supplementary Figures and Tables

1.1 Supplementary Figures

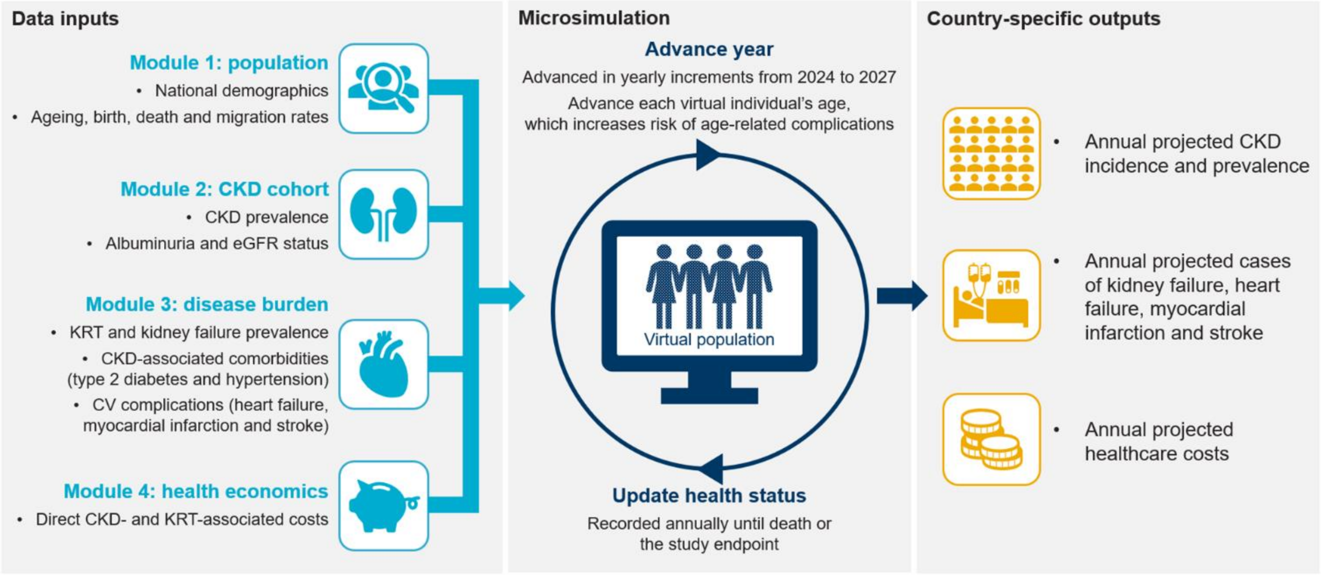

Supplementary Figure 1. Overview of the structure of the *Inside CKD* microsimulation model. CKD, chronic kidney disease; CV, cardiovascular; eGFR, estimated glomerular filtration rate; KRT, kidney replacement therapy. © CC BY-NC 2022. Figure adapted from: Projecting the Epidemiological and Economic Impact of Chronic Kidney Disease Using Patient-Level Microsimulation Modelling: Rationale and Methods of *Inside CKD*. N. Tangri *et al.* (Figure 2). https://doi.org/10.1007/s12325-022-02353-5. This work is licensed under a Creative Commons Attribution-NonCommercial 4.0 International License http://creativecommons.org/licenses/by-nc/4.0/.

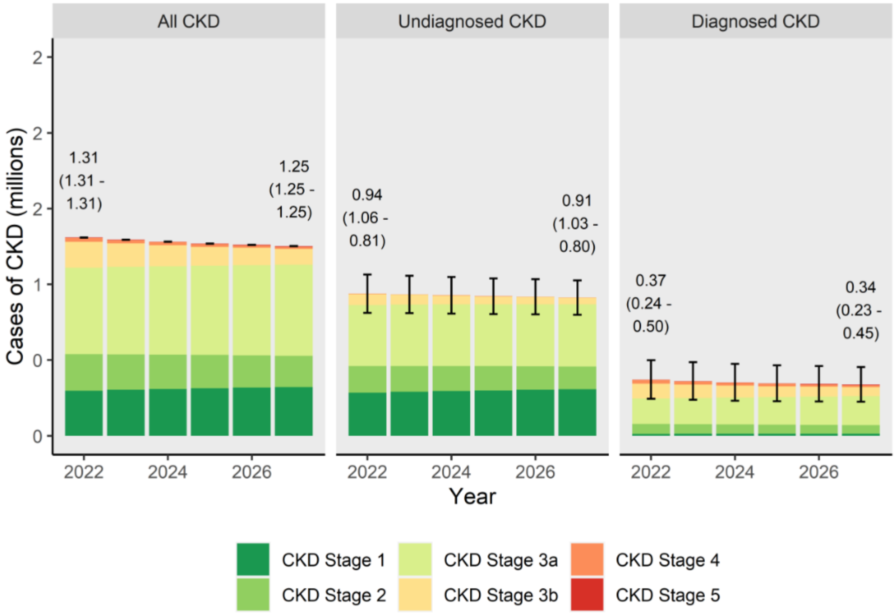

Supplementary Figure 2. Prevalence of CKD following sensitivity analysis of diagnosis rates in which an absolute 10% increase and a 10% decrease were applied to the diagnosis rate. Analysis by total, undiagnosed and diagnosed CKD are provided. The error bars represent the top and bottom estimates of CKD prevalence for a ±10% difference in diagnosis rate. CKD, chronic kidney disease. © CC BY 2024. Projecting the clinical burden of chronic kidney disease at the patient level (*Inside CKD*): a microsimulation modelling study. G. Chertow *et al.* (Supplementary data appendix 1: sensitivity analysis on diagnosis rates). https://doi.org/10.1016/j.eclinm.2024.102614. This work is licensed under a Creative Commons Attribution License https://creativecommons.org/licenses/by/4.0/.

1.2 Supplementary Tables

Supplementary Table 1. Definitions of CKD stages 1–5 used for the *Inside CKD* microsimulation.

	**AER**		
**GFR** (mL/min/1·73 m^2)	A1 (< 30 mg/24h; normal to mildly increased)	A2 (30–300 mg/24h; moderately increased)	A3 (> 300 mg/24h; severely increased)
≥ 90 (normal; G1)	No CKD	Stage 1	Stage 1
60–89 (mildly decreased; G2)	No CKD	Stage 2	Stage 2
45–59 (mildly to moderately decreased; G3a)	Stage 3a	Stage 3a	Stage 3a
30–44 (moderately to severely decreased; G3b)	Stage 3b	Stage 3b	Stage 3b
15–29 (severely decreased; G4)	Stage 4	Stage 4	Stage 4
< 15 (kidney failure; G5)	Stage 5	Stage 5	Stage 5

AER, albumin excretion rate; CKD, chronic kidney disease; GFR, glomerular filtration rate.

Supplementary Table 2. Clinical and economic data inputs.

Clinical parameters (CKD, KRT, comorbidities of CKD, cardiovascular complications of CKD, mortality and diagnosis rates) have been previously published. © CC BY 2024. Projecting the clinical burden of chronic kidney disease at the patient level (*Inside CKD*): a microsimulation modelling study. G. Chertow *et al.* (Table S2 [Hungary]). https://doi.org/10.1016/j.eclinm.2024.102614. This work is licensed under a Creative Commons Attribution License https://creativecommons.org/licenses/by/4.0/.

Health economic parameters have been previously published and adapted for use here. © CC BY-NC 2023. Global Economic Burden Associated with Chronic Kidney Disease: A Pragmatic Review of Medical Costs for the *Inside CKD* Research Programme. V. Jha *et al.* (Table S3, Table S4, Table S5). https://doi.org/10.1007%2Fs12325-023-02608-9. This work is licensed under a Creative Commons Attribution-NonCommercial 4.0 International License http://creativecommons.org/licenses/by-nc/4.0/.

**Parameter**	**Reference**	**Proxy country (if applicable)**	**Definitions**
**CKD**			
eGFR	Zdrojewski et al., 2015 [1]	Poland	eGFR data by age and two risk categories, disaggregated using UK data
Albuminuria	Chudek et al., 2014 [2]	Poland	Albuminuria data by age and risk category
**KRT**			
KRT initiation threshold	Pyart et al., 2020 [3]	UK	
Chance of being treated	O'Callaghan et al., 2011 [4]	UK	
Peritoneal dialysis	1. Calculated using NHIFA real-world data<br>2. MANET - Hungarian Society of Nephrology 2016–2019 [5]<br>3. NHIFA real-world data (data on file)<br>4. Analysis from national inpatient claims database (data on file)	Romania (annual risk of transplant failure only)	1. Incidence and prevalence of patients receiving KRT who are undergoing peritoneal dialysis and haemodialysis<br>2. Prevalence of KRT per 100 000 by year<br>3. Transplantation rate per 100 dialysis patient-years<br>4. Annual risk of transplantation failure
Haemodialysis			
Transplantation			
**Comorbidities of CKD**			
Type 2 diabetes	Analysis from national inpatient claims database (data on file)	Romania	Proportion of type 2 diabetes and hypertension in the CKD population, by albuminuria level
Hypertension			

**Cardiovascular complications of CKD**			
Heart failure	Hungarian NHIFA analysis, 2019 (data on file)		Incidence and prevalence
	USRDS, 2009 [6]	USA	Relative risk
Myocardial infarction	Hungarian NHIFA analysis, 2019 (data on file)		Incidence and prevalence
	USRDS, 2009 [6]	USA	Relative risk
Stroke	Hungarian NHIFA analysis, 2019 (data on file)		Incidence and prevalence
	Masson et al., 2015 [7]		Relative risk
**Mortality**			
Mortality by CKD stage	UN, World Population Prospects, 2019 [8, 9]		Total number of deaths by age and sex
	Levey et al., 2011 [10]		Probability of dying from any cause by CKD stage
**Diagnosis rates**			
Diagnosis rates by stage	Hirst et al., 2020 [11]	UK	Stages 1 and 2
	Average from TriNetX and LCED database analysis by AstraZeneca (data on file)	USA	Stages 3a and 3b
	Bakris et al., 2020 [12]	USA	Stages 4 and 5
**Health economics**			
CKD stage 3a	Analysis of Hungarian NHIFA database, 2022 (data on file)		Patients who had at least one relevant ICD-10 code reported in the payer's database which was retrospectively analysed using the Hungarian NHIFM data
CKD stage 3b			
CKD stage 4			
CKD stage 5 (pre-KRT)			
Haemodialysis			Included labour, medication, central expenditure, inpatient stay expenses (e.g. laundry, food), intensive care unit usage, operating theatre use, emergency care, pharmacy provisioning, and overheads
Peritoneal dialysis	NEAK curative-preventive care database [13]		
Transplant	Analysis of Hungarian NHIFA database, 2022 (data on file)		

Transplant maintenance	State Audit Office of Hungary, 2012		Average cost of maintenance of year 1 and year 2+ based on the state audit office of Hungary report; 9/1993 NM degree. DRG and ICPM database
Heart failure	Analysis of Hungarian NHIFA database, 2022 (data on file)		Inpatient costs include DRG financing, ratio x base fee, regulated in 9/1993 decree. Outpatient costs include that of an intervention, point value x base fee; pharmacy drug: public reimbursement costs
Stroke	Lorenzovici et al, 2020, [14]	Romania	Inpatient hospital costs
Myocardial infarction	Analysis from national inpatient claims database (data on file)	Romania	Includes labour, medication, central expenditure, impatient stay expenses (laundry, food etc.), intensive care unit usage, operating theatre use, emergency care, pharmacy provisioning, and overheads
GDP	The World Bank, 2021		Year 2021
Total healthcare expenditure	Hungarian State Treasury (data on file)		Total national public healthcare expenditures in 2019

CKD, chronic kidney disease; DRG, diagnosis-Related-Group financing system; eGFR, estimated glomerular filtration rate; GDP, gross domestic product; ICD, International Classification of Diseases; ICPM, International Classification of Procedures in Medicine; KRT, kidney replacement therapy; LCED, Explorys Linked Claims and Electronic Medical Records Data; MANET, Magyar Nephrologiai Társaság (Hungarian Nephrology Society); NEAK, Nemzeti Egészségbiztosítási Alapkezelő (National Health Insurance Fund); NHIFA, National Health Insurance Fund Administration; NHIFM, National Health Insurance Fund Manager; UN, United Nations; USRDS, United States Renal Data System; WHO, World Health Organization.

2 Supplementary references

1. Zdrojewski Ł, Zdrojewski T, Rutkowski M, *et al.* Prevalence of chronic kidney disease in a representative sample of the Polish population: results of the NATPOL 2011 survey. *Nephrol Dial Transplant* (2016) 31(3):433–9.

2. Chudek J, Wieczorowska-Tobis K, Zejda J, *et al.* The prevalence of chronic kidney disease and its relation to socioeconomic conditions in an elderly Polish population: results from the national population-based study PolSenior. *Nephrol Dial Transplant* (2014) 29(5):1073–82.

3. Pyart R, Evans K, Steenkamp R, *et al.* The 21st UK Renal Registry Annual Report: A Summary of Analyses of Adult Data in 2017. *Nephron* (2020) 144(2):59–66.

4. O'Callaghan CA, Shine B, Lasserson DS. Chronic kidney disease: a large-scale population-based study of the effects of introducing the CKD-EPI formula for eGFR reporting. *BMJ Open* (2011) 1(2):e000308.

5. Hungarian Society of Nephrology. MANET 2016–2019 (2020) http://www.nephrologia.hu/info.aspx?sp=200 (accessed Aug 01, 2021). [Hungarian].

6. US Renal Data System, USRDS 2009 Annual Data Report: Atlas of Chronic Kidney Disease and EndStage Renal Disease in the United States (2009) https://www.usrds.org/2009/pdf/V1_06_09.pdf. (accessed Aug 01, 2022).

7. Masson P, Webster A, Hong M, *et al.* Chronic kidney disease and the risk of stroke: a systematic review and meta-analysis. *Nephrol Dial Transplant* (2015) 30(7):1162–9.

8. United Nations. Deaths by age groups, male. World population prospects 2019 (2019). https://population.un.org/wpp/Download/Archive/Standard/ (accessed May 4, 2022).

9. United Nations. Deaths by age groups, female. World population prospects 2019 (2019). https://population.un.org/wpp/Download/Archive/Standard/ (accessed May 4, 2022).

10. Levey AS, de Jong P, Coesh J, *et al.* The definition, classification, and prognosis of chronic kidney disease: a KDIGO Controversies Conference report. *Kidney Int* (2011) 80(1):17–28.

11. Hirst JA, Hill N, O'Callaghan C, *et al.* Prevalence of chronic kidney disease in the community using data from OxRen: A UK population-based cohort study. *Br J Gen Pract* (2020) 70(693):e285–93.

12. Bakris G. Prevalence and factors associated with undiagnosed chronic kidney disease in diabetes mellitus (2020) https://casehippo.com/spa/symposium/national-kidney-foundation-2019-spring-clinicalmeetings/event/gallery/abstracts?abstractId=119 (accessed Aug 01, 2021).

13. NEAK curative-preventative care database. Performance data of artificial kidney treatments. (2022) [Accessed: April 2023]; Available from: https://neak.gov.hu/pfile/file?path=/letoltheto/altfin_dok/altfin_virt_dok2/publikus_adatok_gy

me/alapdijak_telj_adatok/A_gyogyito-megelozo_ellatasokra_vonatkozo_kozerdeku_adatok&inline=true.

14. Lorenzovici, L., et al., Cost Assessment of Inpatient Care Episodes of Stroke in Romania. *Front Public Health*, (2020) 4(8):e605919.

15. The World Bank. GDP (current US$) – Hungary (2021) [Accessed Aug 01 2021; Available from: https://data.worldbank.org/indicator/NY.GDP.MKTP.CD?locations=HU
